# New insight into strategies used to develop long-acting G-CSF biologics for neutropenia therapy

**DOI:** 10.3389/fonc.2022.1026377

**Published:** 2023-01-05

**Authors:** Abdulrahman Theyab, Khalaf F. Alsharif, Khalid J. Alzahrani, Atif Abdulwahab A. Oyouni, Yousef MohammedRabaa Hawsawi, Mohammad Algahtani, Saad Alghamdi, Amal F. Alshammary

**Affiliations:** ^1^ Department of Laboratory and Blood Bank, Security Forces Hospital, Makkah, Saudi Arabia; ^2^ College of Medicine, Al-Faisal University, Riyadh, Saudi Arabia; ^3^ Department of Clinical Laboratory Sciences, College of Applied Medical Sciences, Taif University, Taif, Saudi Arabia; ^4^ Department of Biology, Faculty of Sciences, University of Tabuk, Tabuk, Saudi Arabia; ^5^ Research Center, King Faisal Specialist Hospital and Research Center, Jeddah, Saudi Arabia; ^6^ Laboratory Medicine Department, Faculty of Applied Medical Sciences, Umm Al-Qura University, Makkah, Saudi Arabia; ^7^ Department of Clinical Laboratory Sciences, College of Applied Medical Sciences, King Saud University, Riyadh, Saudi Arabia

**Keywords:** G-CSF, long-acting, neutropenia, strategy, therapy

## Abstract

Over the last 20 years, granulocyte colony-stimulating factors (G-CSFs) have become the major therapeutic option for the treatment of patients with neutropenia. Most of the current G-CSFs require daily injections, which are inconvenient and expensive for patients. Increased understanding of G-CSFs’ structure, expression, and mechanism of clearance has been very instrumental in the development of new generations of long-acting G-CSFs with improved efficacy. Several approaches to reducing G-CSF clearance *via* conjugation techniques have been investigated. PEGylation, glycosylation, polysialylation, or conjugation with immunoglobulins or albumins have successfully increased G-CSFs’ half-lives. Pegfilgrastim (Neulasta) has been successfully approved and marketed for the treatment of patients with neutropenia. The rapidly expanding market for G-CSFs has increased demand for G-CSF biosimilars. Therefore, the importance of this review is to highlight the principle, elimination’s route, half-life, clearance, safety, benefits, and limitations of different strategies and techniques used to increase the half-life of biotherapeutic G-CSFs. Understanding these strategies will allow for a new treatment with more competitive manufacturing and lower unit costs compared with that of Neulasta.

## Highlights

- Most of the current G-CSFs require daily injections, which are inconvenient and expensive.
- Different strategies have been used to overcome the short half-life of the first-generation rhG-CSFs.
- Understanding of G-CSF structure, expression, and mechanism of action on neutrophils may contribute to development of safe long-acting G-CSF therapies for patients with neutropenia.

## 1 Introduction

In 1991, filgrastim (FIL; NEUPOGEN^®^) was the first recombinant human granulocyte colony-stimulating factor (rhG-CSF) used for hematopoietic progenitor cell (HPC) mobilization. Filgrastim is a non-glycosylated G-CSF form produced in *E. coli*. It has a molecular weight of approximately 18.8 kDa and contains a methionine group at its N-terminus. It was the first G-CSF medicine approved by the United States Food and Drug Administration (US FDA) for treating neutropenia for various indications (1–3). Filgrastim is indicated to decrease the neutrophil recovery time and the duration of fever in patients with acute myeloid leukemia. It can be used in patients with cancer receiving myelosuppressive chemotherapy (MSC) to reduce the incidence of infection and prevent febrile neutropenia (FN). Filgrastim is also used to reduce neutropenia in patients with non-myeloid leukemia who are undergoing myeloablative chemotherapy followed by bone marrow transplantation. In addition, filgrastim has been approved to treat hematopoietic syndrome of acute radiation syndrome (the treatment plan for patients acutely exposed to myelosuppressive doses of radiation). It is the most commonly used growth factor for the mobilization of autologous HPCs into the peripheral blood (4). It has a short half-life (between 3.5 and 3.8 h on average), with filgrastim concentration and neutrophil count being the factors of clearance. The drug is cleared by the kidney (5–8).

Lenograstim is another first-generation rhG-CSF. It is produced from Chinese hamster ovary cells and has a single *O*-glycosylated form at position Thr-133 (9). It also has a short half-life similar to that of filgrastim, with the *O*-linked providing stability by shielding the cysteine-17–containing sulfhydryl group from oxidation by free radicals (5, 10). It was assumed that lenograstim may show clinical benefits over filgrastim, although an *in vivo* comparative study exhibited no differences between the two rhG-CSF products (11).

Tbo-filgrastim is another short-acting G-CSFs that produced by recombinant DNA technology using the *E. coli* K802 bacterium strain. It is a non-glycosylated recombinant methionyl human granulocyte colony-stimulating factor. It is composed of 175 amino acids and has a molecular weight of approximately 18.8 kDa. Tbo-filgrastim was approved by the European Union as a biosimilar to filgrastim in 2008. Four years later, US FDA had approved tbo-filgrastim as a biologic product with one similar indication to filgrastim (12, 13).

To overcome the short half-life of the first-generation rhG-CSFs, different strategies have been employed to increase G-CSF half-life, which include increasing the molecular weight by conjugation with another moiety such as glycosylation, polysialation, and PEGylation to overcome rapid elimination by renal filtration. In addition, using the mechanism of neonatal fragment crystallizable (Fc) receptor (FcRn) recycling through fusing several proteins with Fc portion of albumin or immunoglobulin (14–16) (**Table 1**). These strategies have successfully prolonged G-CSF half-life (40). This article reviews the underlying principles, elimination’s route, half-life, clearance, safety, benefits, and limitations of each of these strategies from a chemical and structural standpoint.

**Table 1:** Long-acting G-CSF formulations are considered for increasing the *in vivo* residence time of G-CSF compared with native G-CSF, filgrastim (rhG-CSF), and Neulasta.

Drug	Technology (specific modification)	Subcutaneous elimination (half-life)	Stage of development	References
Maxy-G34	Pegylated G-CSF with 5-kDa PEG residue attached to 3 amino acids	Exhibited a median half-life approximately 2.3 times that of Neulasta	Completed phase IIa	(17)
Mecapegfilgramtim	Covalently bonding a 19-kDa polyethylene glycol (PEG) to the N terminus of filgrastim	55.99 h	Completed phase III	(18–21)
Leridistim	Leridistim reacted with 30,000-MW methoxy-peg-propionaldehyde (M-peg-ALD)	Elimination half-life from 7.8 to 33 h	Completed phase III	(18, 22–24)
Lonquex Lipegfilgrastim	A recombinant methionyl hG-CSF which is altered at the natural *O*-glycosylation site (i.e., threonine 134) using a technology of novel glycoPEGylation.	The average terminal half-life ranged from approximately 32 to 62 h	phase III	(25, 26)
Tandem molecule	G-CSF–glycosylated linkers-G-CSF	(approximatly 6–10 h intravenously injected) three-fold longer circulating half-life compared with native G-CSF (1.79 h)	Preclinical	(27, 28)
StimuXen (Lipoxen)	Polysialation (attach poly Sialic acid to N-terminal of G-CSF).	———–	Preclinical	(29)
3DHSA-G-CSF	Domain III of human serum albumin fused to G-CSF	Serum half-life (3.425 ± 0.098 h) in comparison with native G-CSF (2.071 ± 0.037 h)	Preclinical	(30)
ABD-GCSF	Albumin-binding domain (ABD) fused to N terminal end of G-CSF	Serum half-life (9.3 ± 0.7 h) in comparison with Filgrastim (1.7 ± 0.1 h)	Preclinical	(31)
Balugrastim	Fusion of G-CSF N-terminal to the C-terminal of recombinant serum human albumin	Median terminal elimination half-life ≈37 h compared with pegfilgrastim (≈45 h)	Completed phase III	(18, 32–34)
G-CSF/IgG-Fc and G- CSF/IgG-CH	Fusion of G-CSF to IgG1 and IgG4 (Fc and CH domains), respectively.	A longer circulating half-life (five- to eight-fold) than native G-CSF	Preclinical	(35)
Eflapegrastim (Rolvedon)	Fusion of G-CSF to the human IgG4 Fc fragment *via* a 3.4-kDa PEG	36.4 h and has greater potency than pegfilgrastim in chemotherapy-induced neutropenic rats	Approved by the FDA	(36–39)
Pegfilgrastim (Neulasta^®^)	20-kDa PEG molecule is linked to the recombinant N-terminal methionine (r-met) residue of rhG-CSF Neupogen (Filgrastim)	15 up to 80 h	Approved by the FDA	(8)

## 2 Increasing the molecular weight of G-CSF to extend its half-life

### 2.1 Polyethylene glycol

**Figure 1:**
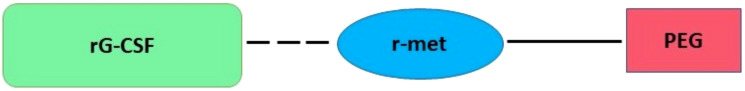
Plan structures of pegfilgrastim. Covalently bound of a single 20-kDa PEG molecule attached to the recombinant N-terminal methionine (r-met) residue of rG-CSF.

Conjugation with polyethylene glycol (PEG), known as PEGylation, was first described in the 1970s by both Abuchowski and Davis, who discovered that PEGylation process may increase the longevity of different proteins and improve their immunological properties, such as albumin and bovine liver catalase (41). Consequently, studies have been performed with a view to improving the PEG process, resulting in PEGs with a wide range of molecular weights (42). In 2003, a notable example is pegvisomant (recombinant pegylated growth hormone antagonist), which was used to treat patients with acromegaly (43, 44). Pegfilgrastim (Neulasta^®^) is another PEGylated form of second-generation rhG-CSF, and it is the only long-acting (once weekly) G-CSF that has been approved by the FDA (**Figure 1**) (6, 45).

#### 2.1.1 Pegfilgrastim (Neulasta^®^)

A single 20-kDa PEG molecule is covalently bound to the recombinant N-terminal methionine (r-met) residue of rhG-CSF Neupogen (Filgrastim) (**Figure 1**) (6, 45). Structurally, each ethylene oxide of PEG can combine with two or three water particles, increasing its water solubility (more hydrophilic) and hydrodynamic radius. This increased molecule size to ~38.8 kDa, consequently decreasing renal clearance. Moreover, PEG technology generates a hydrophilic protection that shields the proteins from immunologic recognition and proteolysis (46, 47).

##### 2.1.1.1 Route of elimination, half-life, clearance, and safety

The addition of a PEG moiety to rhG-CSF reduces its renal clearance *via* glomular filtration, making neutrophil-mediated clearance the primary route of elimination (8). This elimination route is started when pegfilgrastim binds to the G-CSF receptor on the surface of neutrophil cells, causing the pegfilgrastim–receptor complex to be internalized through endocytosis and then degraded inside the cell (48). Following subcutaneous administration, the serum half-life of pegfilgrastim varies hugely depending on the absolute neutrophil counts, with a range of 15 to 80 h. Forty-two hours is the median serum half-life (8).

Pegfilgrastim has a neutrophil-induced self-regulating clearance mechanism (48, 49). The clearance is dependent on the neutrophil counts and body weights of the patients; the clearance increases with an increasing number of granulocytes and lower body weights (FDA-approved drug products). Following chemotherapy-induced neutropenia, pegfilgrastim remains in the blood until neutrophils begin to recover; as neutrophil numbers increase, pegfilgrastim’s elimination increases (49). The obvious clearance of serum is 14 ml/h/kg (Cancer Care Ontario Drug Information: Pegfilgrastim).

The highest dose of pegfilgrastim reported in clinical trials was 300 mcg/kg (50). Pegfilgrastim’s overdosage may result in bone pain and leukocytosis. In case of overdose, patients should be observed for signs and symptoms of toxicity and given general supportive care as necessary (50, 51).

##### 2.1.1.2 Benefits and limitations

The advantage of pegfilgrastim over filgrastim was its ability to avoid renal clearance. However, it is still cleared *via* neutrophil-mediated clearance (52), which is reliant on the number of circulating neutrophils. Hence, pegfilgrastim’s concentration remains high in serum during neutropenia and begins to clear once the neutrophil starts to normalize. Therefore, a single dose of pegfilgrastim is equal to seven daily injections of filgrastim (53).

In addition to the high cost of PEGylated proteins (54), they are usually excreted through the renal system without undergoing primary biodegradation that causes renal toxicity (55) as evidenced by the presence of PEG in bile (56) and vacuoles in renal tubules (29). It has been shown that PEGylation reduced the *in vitro* bioactivity of rhG-CSF to two- or three-fold, mainly due to the structural changes in the macromolecule that could attenuates its potency (57, 58). Furthermore, administration of first dose of PEGylated proteins could induce the production of anti-PEG immunoglobulin M (IgM). However, upon the administration of subsequent doses, liver Kupffer cells start to eradicate these harmful IgMs (59).

### 2.2 Glycosylation

Glycosylation is a common enzymatic modification and refers to the process of adding glycans to macromolecules (60). Glycosylation increases the molecular weight of proteins, improves thermal stability, and prevents proteolytic degradation. The cell membrane surface of glycoproteins contains sialic acid, which induces the overexpression of negatively charged monosaccharides, thereby inhibiting the passage of glycoproteins *via* charge repulsion alongside the glomerular filtration membrane of the kidney and extending their circulatory half-life (61, 62).

Glycosylation might help to reduce the immunogenicity of polypeptides by enhancing their solubility, shielding the hydrophobic residues, and decreasing the possibility of aggregation that may result of a stationary precipitate for antibody recognition (63). An additional proposed theory suggests that sialylation (sialic acid located at the terminal of glycan) shields peptides by reducing the visible surface area that is exposed to antibody recognition sites. Darbepoetin alfa was the first to report this mechanism (a recombinant human erythropoietin analogue consisted of two *N*-linked glycosylation sequences) (64, 65).

Different classes of protein glycosylation have been identified, such as the addition of *N*-linked glycans, *O*-linked glycans, glycosam inoglycans, phosphorylated glycans, and formation of glycosylphosphatidylinositol anchors to peptide backbones as well as residues of C-mannosylation-tryptophan (60). However, the two major forms of glycosylation associated with G-CSF are *N*-linked and *O*-linked glycosylation (66). Understanding the forms and functions of the glycosylation to G-CSF is essential for an optimal conjugation of glycosylation moieties to G-CSF, as evidenced in lipegfilgrastim development.

*O*-linked glycosylation is a diverse protein glycan that is naturally attached to the oxygen atom of hydroxyl groups (-OH) of serine (Ser), threonine (Thr), or tyrosine (Tyr) residues within a protein (60, 67). Nevertheless, no specific consent sequences or motifs have been documented for this attachment. Furthermore, it is unknown why certain Ser/Thr residues are oppositely glycosylated to other residues. It is possible that the protein’s alternative structure contributes to the glycosylation site’s availability (64). The *O*-glycan biosynthetic pathways start in the Golgi apparatus, where polypeptide GalNAc transferase (GlcNAcT) catalyzes the transfer of the GalNAc moiety of uridine diphosphate to the hydroxyl of Ser or Thr (68). Later, a few glycosyltransferases can convert the resulting glycoprotein into different core structures linked by different α- or β-glycosidic linkages (69). *O*-linked glycosylation is found at Thr-134 site of rhG-CSF and recognizable as the only site altered with a single mannose, allowing glycoengineered Pichia. Pastoris is used as a possible model for biotherapeutic rhG-CSF production (70).

**Figure 2:**
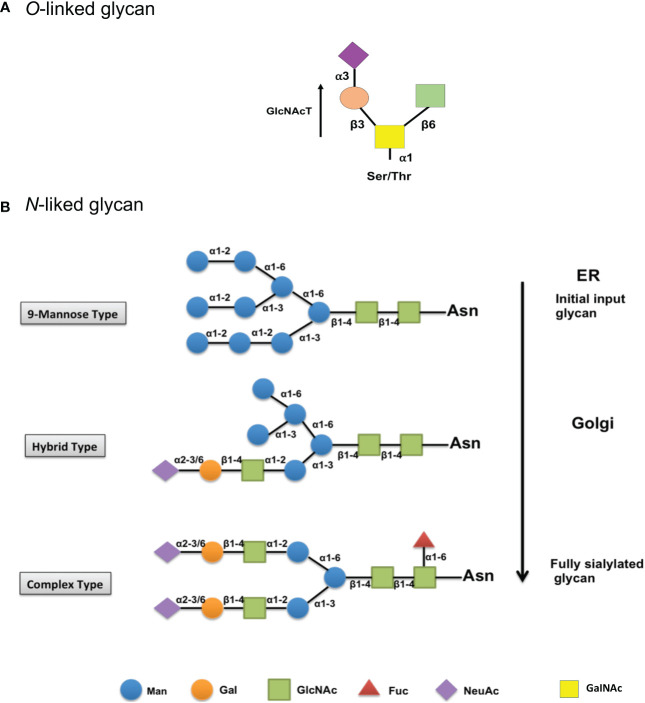
Biosynthesis and structure of *O*- and *N*-linked glycans. **(A)** Initial biosynthetic pathway of *O*-glycans with an *O*-GalNAc moiety starts in the Golgi apparatus, where a GalNAc residue is attached by different polypeptide polypeptide GalNAc transferases (GlcNAcT). Later, a few glycosyltransferases can convert the resulting glycoprotein into different core structures. **(B)** The biosynthetic pathway of the early input glycan (9-mannose glycan) begins in the endothelial reticulum (Top). Then, glycoprotein migrates to the Golgi, where the mannose group is removed, and other monosaccharides are added in a hybrid type process (mid). The biosynthetic process is then finished as a completely sialylated glycan complex in the Golgi (Bottom). Man, mannose; Gal, galactose; GlcNAc, *N*-acetylglucosamine; Fuc, fucose; NeuAc, sialic acid; GalNAc, *N*-acetylgalactosamine.

In *N*-linked glycosylation, an *N*-acetylglucosamine (GlcNAc) residue attaches to the amide group of an asparagine residue. It occurs in consensus sequences Asn-X-Ser/Thr, where X indicates any residue except proline (Pro) (71). *N*-glycosylation has also been observed at non-canonical motifs in some publications, many of which were found in the conformation Asn-X-Cys (cysteine) (72). *N*-glycosylation biosynthetic pathways start in endothelial reticulum (**Figure 2**). An early attachment of a 9-mannose glycan to the peptides of an *N*-linked glycan identifies it as a high mannose type. The inclusion of *N*-glycans is critical for the folding of newly synthesized proteins to be regulated. Following the effective folding of newly synthesized proteins, the glycoprotein migrates to the Golgi apparatus, where mannosidases remove the mannose sugar group. Later, specific glycosyltransferases assist in the binding of various monosaccharides into a developing glycan chain. This hybrid type has a high mannose content (73). The biosynthetic process in the Golgi apparatus is now complete, with a fully sialylated glycan complex containing six sugars: mannose (Man), galactose (Gal), *N*-acetylglucosamine (GlcNAc), fucose (Fuc), sialic acid (NeuAc), linked by different α- or β-glycosidic linkages (recognized as a complex type) (74, 75).

The *N*-linked glycan terminal site contains sialic acid (*N*-acetylneuraminic acid, NeuAc), characterized by a diverse group of nine carbon-containing carbohydrates with a negatively charged carboxylate (C1), and has been shown to be critical in maintaining the half-life of several glycoproteins in the bloodstream (74, 76, 77). Sialic acids can confer glomerular filtration or blood cell charge repulsion due to their negative charge and hydrophilicity, which may delay glycoproteins in the blood circulation as evidenced in human erythropoietin (78, 79).

#### 2.2.1 Application of *N*-linked glycan in G-CSF drug development

*O*-linked glycosylation occurs most commonly at clustered Ser or Thr residues, making *N*-glycan profiling more feasible than *O*-glycan profiling, as evidenced by a universal endoglycosidase. Peptide-*N*-glycosidase F catalyzes the deglycosylation of most *N*-glycans and cleaves 9-high-mannose, hybrid, and complex monosaccharide chains but has not been identified for *O*-glycan (80, 81). Hence, *N*-linked glycan is preferred in several technologies of protein modification and biopharmaceutical functioning (82, 83). Recent strategies of *N*-linked glycan can be classified into site-directed mutagenesis and glycosylated linker.

##### 2.2.1.1 Site-directed mutagenesis (glycoengineering)

Glycoengineering is a process of enhancing the properties of therapeutic proteins through altering their glycosylation to improve its pharmacokinetic and biological activity (84). One of the most common methods for glycoengineering is DNA mutagenesis. *In vivo*, additional glycosylation sites can be added to DNA *via* mutagenesis. This can be performed, for example, by detecting the third position of Thr/Ser residues in a protein’s sequence and mutating the first amino acid to Asn or by detecting Asn residues in a protein’s sequence and mutating the third amino acid to Thr/Ser (85). Site-directed DNA mutagenesis was used to generate new darbepoetin alfa, by mutating Ala-30, His-32 to Asn-30, Thr-32, Pro-87, Trp-88, Pro-90 to Val-87, Asn-88, and Thr-90. Alterations were found to be glycosylated, with a molecular weight increase from 35 to approximately 43 kDa while retaining biological activity (86). G-CSF has also been performed to site-directed mutagenesis by mutating Phe-140 to Asn-140, resulting in an *N*-linked glycosylation site on rhG-CSF. This novel mutant was exhibited to be glycosylated and had more efficiency at stimulating hematopoietic cell proliferation and differentiation than native G-CSF (16).

##### 2.2.1.2 Glycosylated linker (tandem molecules)

The use of glycosylated linker is another approach for increasing longevity and enhancing bioactivity of some therapeutic proteins in serum. Glycosylated linkers have been shown to be incorporated between two ligands of the same protein, as demonstrated in the development of recombinant human follicle-stimulating hormone (rhFSH), where either an *O*- or *N*-linked glycosylated linker was used between the α and β subunits of rhFSH. As a result, glycosylated linker increased half-life of the new tandem rhFSH by up to two-fold compared with native FSH (87, 88).

**Figure 3:**
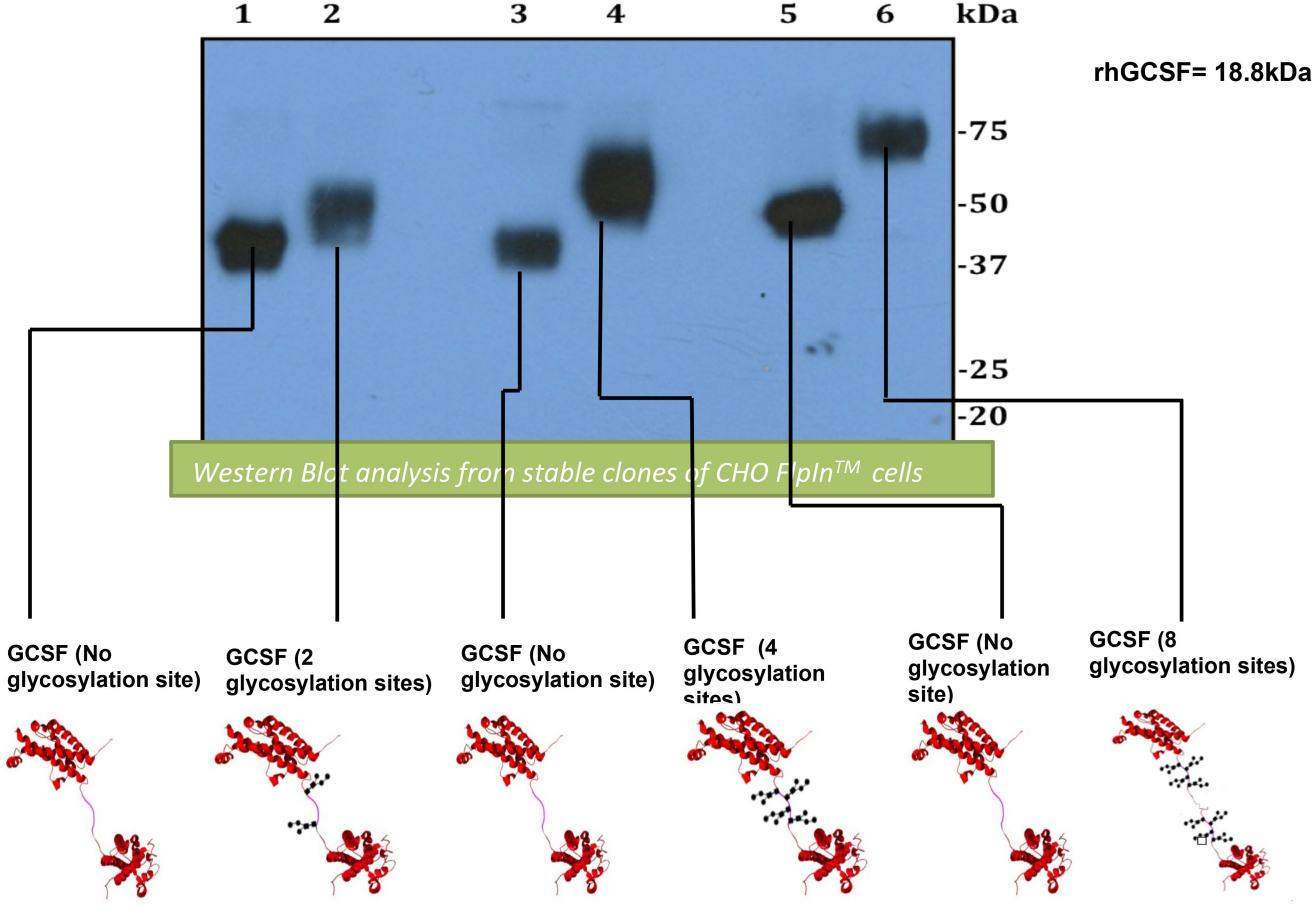
Analysis of Western blot expressing and comparing G-CSF tandems and their controls. First lane, GCSF2QAT_control (2 × QAT). Second lane, GCSF2NAT (2 × NAT). Third lane, GCSF4QAT_control (4 × QAT). Fourth lane, GCSF4NAT (4 × NAT). Fifth lane, GCSF8QAT_control (8 × QAT). Sixth lane, GCSF8NAT (8 × NAT). Analysis of Western blot displays an obvious increase in molecular weight for glycosylated GCSF tandems (GCSF2NAT, GCSF5NAT, and GCSF8NAT) when compared with non-glycosylated controls (GCSF2QAT, GCSF4QAT, and GCSF8QAT).

To generate a long-acting G-CSF, the advantages of glycosylated linker design have been used. Two G-CSF ligands were linked *via* a flexible (Gly4Ser)n linker including different glycosylation sites to form a tandem [G-CSF contains two glycosylation motifs (G-CSF2NAT): G-CSF4NAT and G-CSF8NAT] and their respective controls (QAT instead of NAT; Q = glutamine, therefore, is not recognized by cell for glycosylation). Using Western blot, the preclinical study demonstrated an increase in the molecular weight of isolated glycosylated G-CSF tandems compared with controls (**Figure 3**). In comparison to rhG-CSF, all G-CSF tandems exhibit increased bioactivity with two- to three-fold lower half maximal effective concentration (EC50s) (27). After intravenous injection to rats, G-CSF2NAT, G-CSF4NAT, and G-CSF8NAT, including two, four, and eight glycosylation sites, respectively, exhibited a lower rate of clearance in comparison to rhG-CSF (achieved a longer circulating half-life, nearly three-fold compared with rhG-CSF) (27). Although tandem G-CSF is still in the preclinical stage, we point out that the use of glycosylated linkers is safer than the use of site-directed mutagenesis to avoid mutating G-CSF itself, which may affect the protein’s bioactivity.

#### 2.2.2 Application of *O*- linked glycan in G-CSF drug development

##### 2.2.2.1 Lipegfilgrastim (Lonquex)

**Figure 4:**
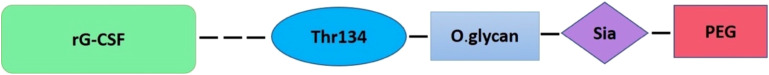
Plan structures of lipegfilgrastim. Conjugation of a single 20-kDa PEG–sialic acid (Sia) to a natural *O*-linked glycan moiety bound at the Thr-134 residue site of rG-CSF.

Lipegfilgrastim is novel long-acting G-CSF, site-specific glycolpegylated r-metHu G-CSF formed by conjugation of a single 20-kDa PEG–sialic acid (Sia) to the *O*-linked glycan bound at the Thr-134 residue site of G-CSF, using the technology of glycopegylation (**Figure 4**) (25, 89). Lipegfilgrastim provides a therapeutic alternative to pegfilgrastim but has more limited global distribution (obtainable in Europe) and, therefore, less experience with its use. Clinical trials of pegfilgrastim have been shown to have a favourable competence and safety profile for prophylactic usage in patients with cancer receiving chemotherapy and at risk of severe neutropenia and may be preferred by both physicians and patients over short-acting G-CSF due to enhanced adherence and a simple once-per-cycle subcutaneous injection (90–92). Lipegfilgrastim was found to be an effective option for reducing the duration of severe chemotherapy-induced neutropenia and preventing FN in older patients with aggressive B-cell NHL receiving MSC (36). Although the current studies published for lipegfilgrastim are still limited, there is an indication that it is a promising treatment for chemotherapy-induced neutropenia.

##### 2.2.2.1.1 Route of elimination, half-life, clearance, and safety

Lipegfilgrastim has two different clearance pathways: a linear pathway involving proteolytic enzyme degradation and a non-linear pathway involving neutrophil-mediated clearance (93). However, at higher doses, the elimination pathway by neutrophil-mediated clearance is saturated, and its degraded fragments may undergo renal clearance (26, 94). After a single subcutaneous injection of 6 mg of lipegfilgrastim in healthy individuals, the average terminal half-life ranged from 32 to 62 h, which was 7–10 h longer for lipegfilgrastim 100 mcg/kg compared with that reported for pegfilgrastim at 100 mcg/kg (26). In phase I of a different multinational, open-label, single-arm study of pediatric patients with the Ewing family of tumors or rhabdomyosarcoma treated with MSC, the average evident clearance (CL/F) was nearly 70 ml/h for patients aged 2–6 years, 120 ml/h for patients aged 6–12 years, and 116 ml/h for patients aged 12–18 years (94).

In studies on the safety of lipegfilgrastim in dogs and rats, a single subcutaneous dose of 10 mg/kg was well tolerated. Similarly, an intravenous dose of 250 mcg/kg was well effective and tolerated in the renal excretion of rats. In a study of 139 patients, adverse events related to lipegfilgrastim occurred in 55 (39.5%) patients; bone pain and back pain were the most common (95).

##### 2.2.2.1.2 Benefits and limitation

It appears that glycolpegylation alters the pharmacokinetic and pharmacodynamic profiles of lipegfilgrastim. In phase I studies involving healthy volunteers, lipegfilgrastim at subcutaneous dose of 6 mg demonstrated 64% greater cumulative exposure and 36% greater peak exposure than pegfilgrastim with the same dose. In addition to pegfilgrastim, lipegfilgrastim had a longer half-life (geometric means, 32.4 h *vs*. 27.2 h, respectively). In a randomized, double-blind, phase III trial, it was determined that lipegfilgrastim was non-inferior to pegfilgrastim in terms of the duration of severe neutropenia in patients with breast cancer (96, 97). Back pain and bone pain were the only limitations, as mentioned previously.

## 3 Prolonged the serum half-life of G-CSF using neonatal Fc receptor

One of the most common antibodies found in extracellular fluids and circulation is immunoglobulin G (IgG). Although it can directly protect the body from infections by activating its antigen binding site, IgG immune functions are mostly mediated through receptors and proteins expressed by special cell subsets that bind to a region of IgG called Fc. The neonatal FcRn belongs to a large family of Fc gamma (γ) receptors (FcγRs) and has become increasingly important on binding IgGs and albumin. FcRn is known as a recycling receptor and has been shown to bind and maintain IgGs and albumin in the blood circulation and to bidirectionally transport both ligand molecules through polarized cellular barriers (98).

**Figure 5:**
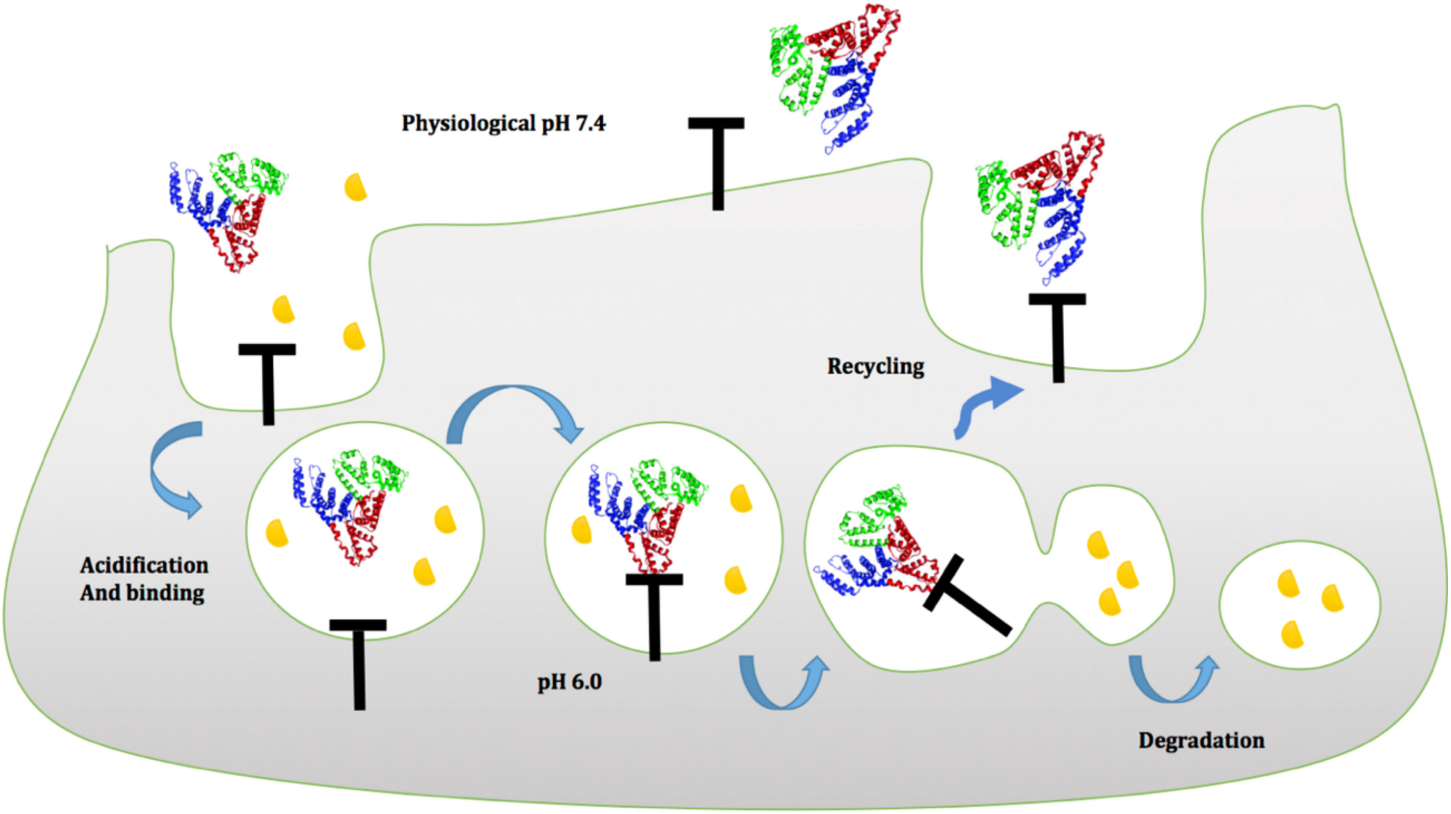
Proposed model of albumin to FcRn in a pH-dependent manner at the cell membrane. Green (domain I), blue (domain II), and red (domain III) indicate the three domains of albumin. Domain III albumin binds to the FcRn receptor at an acidic pH of 6.0, protecting it from lysosomal destruction, and then recycles to the blood circulation, where it is released when exposed to physiological pH 7.4.

In general, FcRn can attach to albumin and IgGs at the cell membrane in a pH-dependent way. Because of the presence of histidines in albumin and IgGs, the imidazole group of histidine protonates at a pH of 6.0 and binds to the FcRn receptor. The FcRn–IgG or FcRn–albumin complex interaction is then internalized and absorbed through the cell membrane, protecting albumin or IgGs from lysosomal degradation (99–101). The FcRn–albumin or FcRn-IgG complex is then recycled to the blood circulation, where it is liberated when exposed to physiological pH 7.4 (**Figure 5**) (102–104).

### 3.1 Application of human serum albumin in G-CSF drug development

Human serum albumin (HSA) is the main protein produced in the liver and serves a variety of physiological functions, including bilirubin, fatty acids, ion transport, and regulation of colloid blood pressure control. Because albumin is a massive protein (molecular weight of 66.5 kDa and an average half-life of 19 days), it can be used to conjugate with multiple recombinant proteins to prolong their half-lives. Consequently, these conjugated proteins will eventually acquire a molecular weight that is too large and challenging for the kidney to filter, extending the residency time of plasma proteins in the blood circulation (100, 105).

Later, the importance of carboxy-terminal domain III human serum albumin (HAS) was recognized and genetically merged with the N-terminus of rhG-CSF. As a result, pharmacokinetic and pharmacodynamic experiments revealed a longer circulating half-life and a high number of white blood cells (WBC) counts in neutropenic mice when compared with rhG-CSF (30).

### 3.2 Fusion of G-CSF to immunoglobulins (Fc and CH domains)

IgG1 and IgG4 immunoglobulins have a circulation half-life of 23 days in serum and have been utilized to generate various long-acting fusion proteins (106). As a result, immunoglobulins were chosen as the ideal antibodies for Fc fusion proteins. They are structurally made up of two identical heavy and light chains that are linked *via* disulfide linkages. Both chains have two regions: the antigen-binding fragment (Fab), an antibody’s head portion, is essential for detecting immunogenicity, whereas the Fc, an antibody’s tail component that interacts with a cell surface receptor, is crucial to maintaining IgG circulation (106).

IgG immunoglobulin is composed of two fragment domains: Fc (Hinge-CH2-CH3) and CH (CH1-Hinge-CH2-CH3). The Hinge domain connects the Fc and Fab areas and allows for more flexibility, and it has been reported that various biotherapeutic proteins can be linked *via* the carboxy-termini of human IgGs’ Fc (Hinge-CH2-CH3) and CH (CH1-Hinge-CH2-CH3) domains (35). IgG fusion proteins are made and released from mammalian cells as disulfide-linked homodimers. This is because cysteine residues in the hinge region of IgGs form inter-chain disulfide bonds with each other. Furthermore, the dimeric structure of IgG fusion proteins increases their effective size and circulation half-life (15, 35).

**Figure 6:**
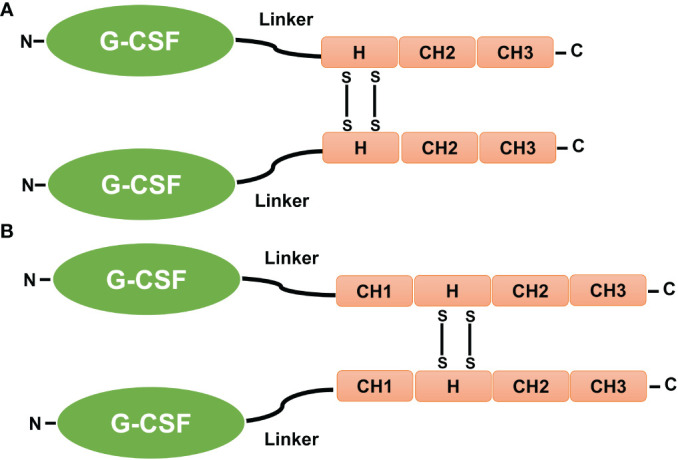
Fusion of G-CSF to Fc and CH domains. **(A)** The carboxy-terminus of human GCSF is fused to the amino termini of the IgG-Fc and IgG-CH domains by a 7–amino acid fixable linker (L). **(B)** The CH1, CH2, and CH3 sections of the IgG domains, as well as the hinge (H), are also shown. The presence of disulfide bonds (SS) that occur between cysteine residues causes fusion proteins in the IgG hinge region to be dimeric (15, 35).

The carboxy-terminus of rhG-CSF has been fused to the amino termini of the Fc (Hinge-CH2-CH3) and CH (CH1-Hinge-CH2-CH3) domains of human IgG4 and IgG1 immunoglobulins, which are connected by a 7–amino acid flexible linker (Ser-Gly-Gly-Ser-Gly-Gly-Ser) (**Figure 6**). Fusions of rhG-CSF to the IgG domain resulted in homodimers with a massive molecular weight, longer circulation half-lives (approximately five- to eight-fold longer than reported for native G-CSF), and a high number of neutrophil counts *in vivo*, without affecting G-CSF bioactivity *in vitro* (15, 35).

#### 3.2.1 Eflapegrastim (*Rolvedon*)

**Figure 7:**
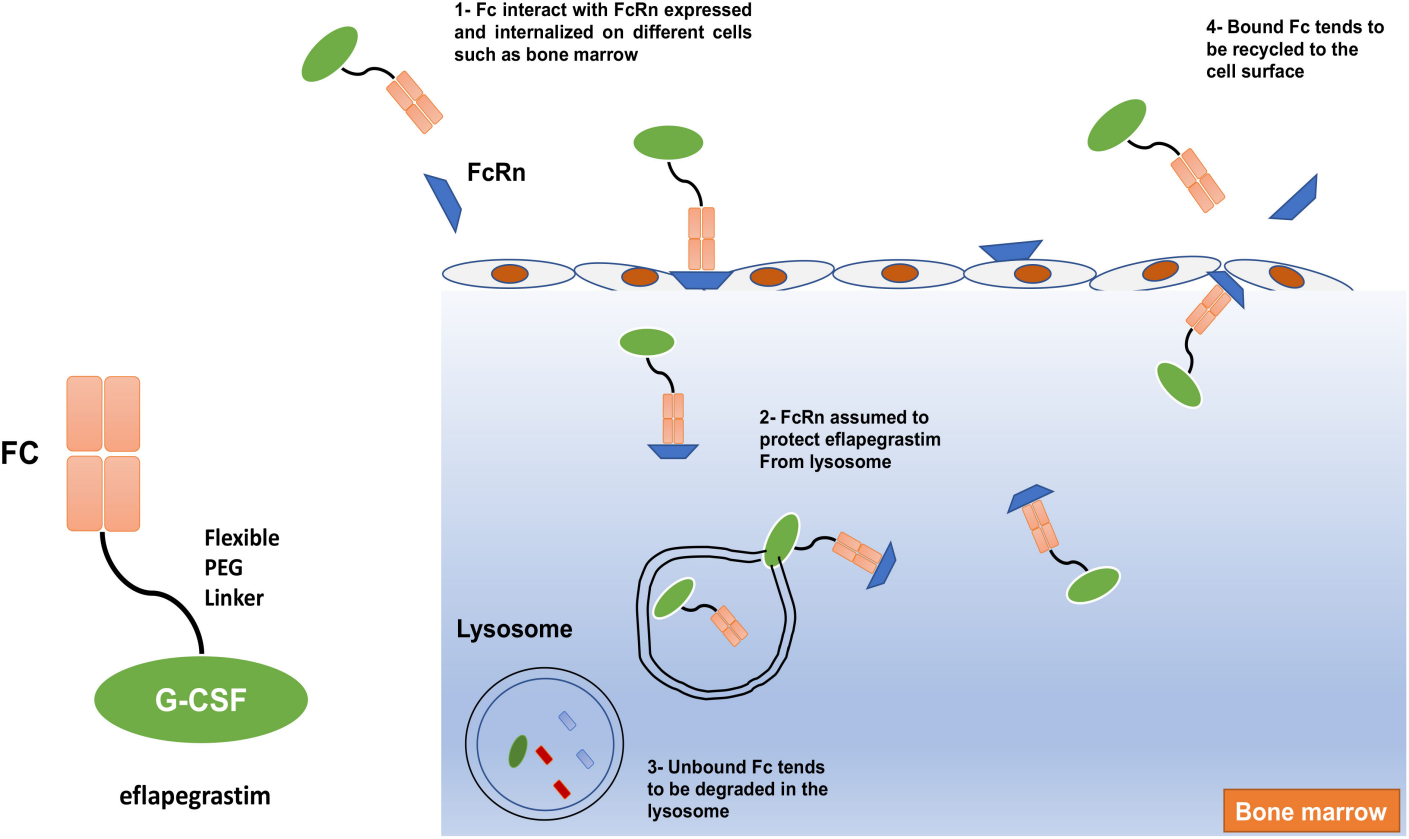
The mechanism of action of eflapegrastim. The human IgG4 Fc fragment of Eflapegrastim interacts with FcRn, which is expressed and internalized on different cells such as bone marrow and assumed to protect eflapegrastim from lysosome leads to elongate its retention in bone marrow.

Eflapegrastim (Rolvedon), a novel long-acting rhG-CSF, is created by fusing the rhG-CSF to the Fc fragment of human IgG4 *via* a PEG linker to increase G-CSF half-life (38, 107). The findings suggested that the human IgG4 Fc fragment of eflapegrastim interacts with FcRn, which is expressed on various tissues including bone marrow, and thus reduces eflapegrastim renal clearance, protects it from lysosomes, and prolongs its retention in bone marrow (**Figure 7**) (108). Preclinical studies of phase I and II pharmacokinetic and pharmacodynamic data exhibited an increased potency for neutrophil count for eflapegrastim compared with pegflgrastim (40, 108, 109). Phase III results showed noninferiority and analogous safety for eflapegrastim at a lower dose of G-CSF compared with pegflgrastim. Therefore, eflapegrastim has a high potential to provide improved clinical benefit and permit more clinical studies in patients at higher risk for contrast-induced nephropathy (37, 38). A recent study by Jeon et al. (2022) showed that eflapegrastim has greater potency than pegfilgrastim in chemotherapy-induced neutropenic rats (39, 110). In September 2022, the US FDA approved eflapegrastim as a prophylactic against infection, as evidenced by FN, in patients receiving certain myelosuppressive anti-cancer drugs.

##### 3.2.1.1 Route of elimination, half-life, clearance, and safety

Eflapegrastim is not detectable in the urine after subcutaneous dose administration (109). In patients with breast cancer, eflapagrastim has a median half-life of 36.4 h. Eflapegrastim clearance decreased with increasing dose, indicating target-mediated clearance by neutrophils. Clearance appears to increase with repeated dosing, possibly due to the subsequent increase of neutrophils in the circulation (FDA-approved drug products).

Leukocytosis and bone pain may result from an eflapegrastim overdose. In this case, patients must be monitored for these adverse effects, and general supportive measures should be taken as required (FDA-approved drug products).

##### 3.2.1.2 Benefits and limitation

A study in rat models showed that eflapegrastim improved clinical benefit and was associated with higher bone marrow and serum concentrations than pegfilgrastim, which resulted in a significantly shorter duration of neutropenia when administered 24 h after chemotherapy compared with pegfilgrastim (111). Leukocytosis and bone pain due to eflapegrastim overdose are considered the only limitations, as mentioned previously (FDA-approved drug products).

## 4 Conclusion

All the long-acting G-CSFs in development rely on pegylation, glycopegylation, conjugation to IgG fragments, or serum human albumin (**Table 1**). Nonetheless, understanding G-CSF structure, expression, and mechanism of action on neutrophils may contribute to the development of a safe long-acting G-CSF therapy for patients with neutropenia that maintains the pharmacodynamic and pharmacokinetic of pegfilgrastim (Neulasta), but with more competitive manufacturing and lower unit costs compared with Neulasta (the cost for Neulasta is $6,417.99* per dose as of 18 August 2021).

## Author contributions

All authors contributed equally to this publication. Conception and design: AT and KFA. Drafting: ATA and KFA. All authors contributed to the article and approved the submitted version.
